# Phase diagram of quantum critical system via local convertibility of ground state

**DOI:** 10.1038/srep29175

**Published:** 2016-07-06

**Authors:** Si-Yuan Liu, Quan Quan, Jin-Jun Chen, Yu-Ran Zhang, Wen-Li Yang, Heng Fan

**Affiliations:** 1 Institute of Modern Physics, Northwest University, Xian 710069, P. R. China; 2 Beijing National Laboratory for Condensed Matter Physics, Institute of Physics, Chinese Academy of Sciences, Beijing 100190, P. R. China; 3 School of Mathematical Sciences, Capital Normal University, Beijing 100048, China; 4 Collaborative Innovation Center of Quantum Matter, Beijing, P. R. China

## Abstract

We investigate the relationship between two kinds of ground-state local convertibility and quantum phase transitions in XY model. The local operations and classical communications (LOCC) convertibility is examined by the majorization relations and the entanglement-assisted local operations and classical communications (ELOCC) via Rényi entropy interception. In the phase diagram of XY model, LOCC convertibility and ELOCC convertibility of ground-states are presented and compared. It is shown that different phases in the phase diagram of XY model can have different LOCC or ELOCC convertibility, which can be used to detect the quantum phase transition. This study will enlighten extensive studies of quantum phase transitions from the perspective of local convertibility, e.g., finite-temperature phase transitions and other quantum many-body models.

Many developments in quantum information processing (QIP)^1^ unveiling the rich structure of quantum states and the nature of entanglement have offered many insights into quantum many-body physics^2^. Because the ground-state wavefunction undergoes qualitative changes at a quantum phase transition, it is important to understand how its genuine quantum aspects evolve throughout the transition^3^,^4^. For the spin chain model, this question has been widely discussed^4^,^5^. Many alternative indictors of quantum phase transitions, for example, entanglements measured by concurrence^3^, negativity^6^, geometric entanglement^7^, and von Neumann entropy^8^,^9^ have been investigated in several critical systems, which have become a focus of attention in detecting a number of critical points. From the viewpoint of quantum correlations, mutual information^10^, quantum discord^11^ and global quantum discord^12^,^13^,^14^ have been used for detecting quantum phase transitions. There are investigations on entanglement spectra^15^,^16^,^17^, fidelity^18^ and local quantum thermal susceptibility^19^ showing their abilities in exploring numerous phase transition points in various critical systems as well.

With the concepts of QIP, these studies have achieved great success in understanding the deep nature of the different phase transitions. The reverse, however, is still unclear and deserves further investigations. Recently, from a new point of view, Cui *et al*. find that the systems undergoing quantum phase transition will also show different operational properties from the perspective of QIP both analytically^20^ and numerically^21^,^22^. For several models, they reveal that the entanglement-assisted local operations and classical communications (ELOCC) convertibility decided by the Rényi entropy^23^,^24^ suddenly changes nearly at the critical point. In ref. 25, the local operations and classical communications (LOCC) convertibility decided by the majorization relation^26^ has also been investigated in the one-dimensional transverse field Ising model^27^. These significant results suggest that not only are the methods of QIP useful as alternative signatures of quantum phase transitions, but also the study of quantum phase transitions can offer interesting insight into QIP.

In this paper, we study the relationship between two kinds of ground-state local convertibility and quantum phase transitions in XY model. The majorization relation studied here is necessary and sufficient for the LOCC convertibility^26^ and Rényi entropy interception is a necessary and sufficient condition for the ELOCC convertibility^28^. In the phase diagram of XY model, LOCC convertibility and ELOCC convertibility of ground-states are presented and compared. Since the one-dimensional transverse field Ising model can be seen as a special case of our model, we promote and widen the results in previous literature. It is shown that different phases in the phase diagram of XY model can have different LOCC or ELOCC convertibility and both of them changes around the phase transition point, which have successfully help us understand the deep nature of the different phases in XY model. On the contrary, it will also benefit many applications such as to detect the quantum phase transition and to estimate the computational power of different phases in quantum critical systems^22^.

## Results

### Local convertibility of ground states of XY model

**Figure 1:**
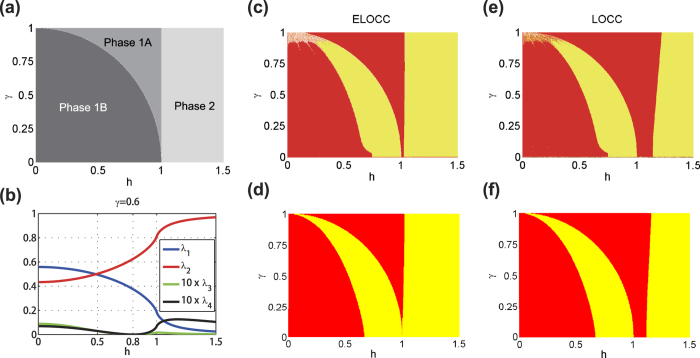
Phase diagram of XY model. (**a**) Phase diagram of XY model. XY model has three different quantum phases: Phase 1A, Phase 1B and Phase 2. (**b**) Eigenvalues of the density matrix of two nearby spins against parameter *h* for *γ* = 0.6. (**c,d**) ELOCC convertibility via Rényi entropy of ground states of XY model. In yellow (light) area, the sign of ∂_*h*_*S*_*α*_(*ρ*_*A*_(*h*)) is negative for all *α* which indicates that |*G*(*h* + Δ)〉_*AB*_ can be transformed to |*G*(*h*)〉_*AB*_ by ELOCC with 100% probability of success. The red (dark) areas are for other cases. (**c**) is obtained by exact diagonalization of the Hamiltonian of XY model, and (**d**) is obtained by the analytical method given in Methods. (**e,f**) LOCC convertibility via majorization of ground states of XY model. In yellow (light) area, |*G*(*h* + Δ)〉_*AB*_ can be transformed to |*G*(*h*)〉_*AB*_ by LOCC with 100% probability of success; whilst, in the red (dark) area, |*G*(*h* + Δ)〉_*AB*_ can not be transformed to |*G*(*h*)〉_*AB*_ by LOCC with certainty. (**e**) is obtained by exact diagonalization of the Hamiltonian of XY model, and (**f**) is obtained by the analytical method given in Methods.

In this section, given the XY model^29^,^30^,^31^ in the zero-temperature case, we use majorization relation and Rényi entropy interception to study the relationship between two kinds of local convertibility and quantum phase transitions. The Hamiltonian of our model is as follows^14^:


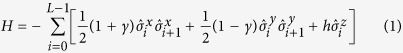


with *L* being the number of spins in the chain, 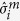 the *i*th spin Pauli operator in the direction *m* = *x*, *y*, *z* and periodic boundary conditions assumed. The XX model and transverse field Ising model thus correspond to the special cases for this general class of models. For the case that *γ* → 0, our model reduces to XX model. The quantum criticality of ground and thermal states in XX Model has been studied^32^. When *γ* = 1, the model reduces to transverse field Ising model^27^. For *h* = 1, a second-order quantum phase transition takes place for any 0 ≤ *γ* ≤ 1. In fact, there exists additional structure of interest in phase space beyond the breaking of phase flip symmetry at *h* = 1. It’s worth noting that there exists a circle boundary between Phase 1A and Phase 1B, *h*^2^ + *γ*^2^ = 1, on which the ground state is fully separable. According to the previous literature, this circle can be seen as a boundary between two differing phases which are characterized by the presence and absence of parallel entanglement^33^,^34^,^35^,^36^,^37^. In fact, for each fixed *γ*, the system is only locally non-convertible when *h*^2^ + *γ*^2^ > 1. Now we can divide the system into three separate regimes, Phase 1A, Phase 1B and Phase 2, where the ferromagnetic region is now divided into two regions defined by their differential local convertibility. These results are summarized in “phase-diagram” Fig. 1(a).

We study the ground states of this model for different system sizes with the field parameter *h* varying from 0 to 1.5. The ground states labeled as 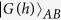 are obtained by exactly diagonalizing the whole Hamiltonian (1). For infinite long spin chain of XY model, the analytical results of the thermal ground states are also discussed, see Methods for details. This proposal is also worth further investigations by other numerical methods such as the Lanczos algorithm and density matrix renormalization group (DMRG), which are not included in this work.

### ELOCC convertibility via entanglement Rényi entropy

Using this method, we provide a phase diagram which describes the ELOCC convertibility for different phases in XY model. We consider the case that Alice shared a reduced system of two spins.

**Figure 2:**
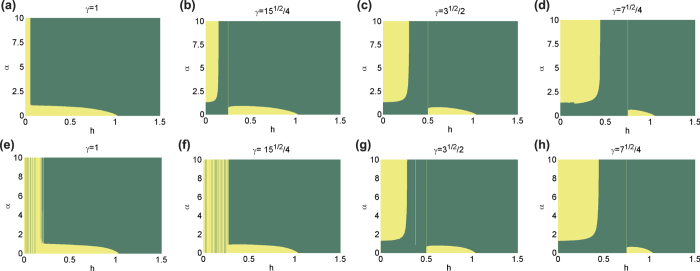
The sign distribution of ∂_*h*_*S*_*α*_(*ρ*_*A*_(*h*)) with Alice having a 2-spin system in XY model. The sign distribution of ∂_*h*_*S*_*α*_(*ρ*_*A*_(*h*)) with Alice having a 2-spin system in XY model for different system size *L* and parameter *γ* against *α* and the adjustable external parameter *h*. The green (dark) areas represent the sign to be negative and the yellow (light) areas indicate positive. In (**a–d**) the system size is *L* = 8, and (**e–h**) show the case that the system size is *L* = 15. Note that the transition between Phase 1A and 2 occurs at *h* = 1, and the transition between Phase 1A and 1B occurs at *h* = 0.25 for 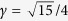 [see (**b,f**)], *h* = 0.5 for 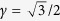 [see (**c,g**)], and *h* = 0.75 for 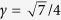 [see (**d,h**)].

In Fig. 1(c), for a system size *L* = 15, we have two different regions that indicate different ELOCC convertibility. In the yellow (light) areas, the sign of ∂_*h*_*S*_*α*_(*ρ*_*A*_(*h*)) is negative for all *α*, that is, the two states |*G*(*h*)〉_*AB*_ and |*G*(*h* + Δ)〉_*AB*_ can be converted to each other by ELOCC. In the red (dark) areas, the sign of ∂_*h*_*S*_*α*_(*ρ*_*A*_(*h*)) is positive for all *α*, there is no ELOCC convertibility in these regions. Similar as Fig. 2(e,f), the obscure areas in Fig. 1(c) reflect the level-crossings that redefine the ground state of the system (which are evident from the spectrum of the model). For infinite long spin chain of XY model, the ELOCC convertibility of the thermal ground state is plotted in Fig. 1(d), see methods for details. Comparing Fig. 1(a) and Fig. 1(c,d), it is obvious that some boundaries between yellow (light) area and red (dark) area can be used to detect the critical line for different phases. Specifically, in Phase 1A, the ELOCC convertibility does not exist; in some area of Phase 1B and the entire area of Phase 2, the ground states |*G*(*h*)〉_*AB*_ and |*G*(*h* + Δ)〉_*AB*_ can be converted to each other by ELOCC. Therefore, the transition between Phase 1A and Phase 2 and the one between Phase 1A and Phase 1B can be described precisely by the ELOCC convertibility via entanglement Rényi entropy.

### LOCC convertibility via majorization relation

For the same case that the reduced system shared by Alice has two spins, we only need to consider three largest eigenvalues of the reduced states of any two neighboring spins as *λ*_1_(*h*), *λ*_2_(*h*), and *λ*_3_(*h*) in descending order. To detect the majorization relations between two ground states |*G*(*h*)〉_*AB*_ and |*G*(*h* + Δ)〉_*AB*_ given some infinitesimal Δ, we should judge the monotonicities of three functions for the field parameter *f*_1_(*h*), *f*_2_(*h*) and *f*_3_(*h*) defined in Eq. (3). Thus, three cases will be met: (*i*) when monotonicities of these three functions are all non-increasing, |*G*(*h* + Δ)〉_*AB*_ can be converted to |*G*(*h*)〉_*AB*_ by LOCC with 100% certainty; (*ii*) when monotonicities of these three functions all are non-decreasing, |*G*(*h*)〉_*AB*_ can be converted to |*G*(*h* + Δ)〉_*AB*_ by LOCC with 100% certainty; (*iii*) except for these two cases, no state can be converted to the other via LOCC with certainty. For ease of comparison with ELOCC convertibility, We only show in Fig. 1(e) either the case (*ii*) or other two cases.

Using the method mentioned above, we can also provide a phase diagram describing the LOCC convertibility for different phase in XY model. In Fig. 1(e), for system sizes *N* = 15, we have shown two different regions. In the yellow (light) area, |*G*(*h* + Δ)〉_*AB*_ can be transformed to |*G*(*h*)〉_*AB*_ by LOCC with 100% probability of success; whilst, in the red (dark) area, |*G*(*h* + Δ)〉_*AB*_ can not be transformed to |*G*(*h*)〉_*AB*_ by LOCC with certainty. We should stress that in few parts of red (dark) area in Phase 1A, |*G*(*h*)〉_*AB*_ can be transformed to |*G*(*h* + Δ)〉_*AB*_ by LOCC with certainty, which is not shown in Fig. 1(e). For infinite long spin chain of XY model, the LOCC convertibility of the thermal ground state is plotted in Fig. 1(f), see methods for details. In the XY model, there are three regimes, Phase 1A, Phase 1B and Phase 2 as shown in Fig. 1(a). From Fig. 1(e,f), in Phase 1A, no majorization relations can be fulfilled, |*G*(*h* + Δ)〉_*AB*_ and |*G*(*h*)〉_*AB*_ can not be converted to each other by LOCC. However, in Phase 1B and Phase 2, there are two areas which have different LOCC convertibility, which have richer and more complex features of LOCC convertibility than those of ELOCC. Similarly, we can use the dividing line between red (dark) and yellow (light) areas with different LOCC convertibility to detect the critical line between Phase 1A and Phase 1B precisely. As the increase of the system size *L*, our result can be shown to get better using the scaling analysis on the critical point from LOCC convertibility^25^ and ELOCC convertibility^21^ to get rid of the finite-size effect.

Comparing the result with the ELOCC method, we can see that the ELOCC method is superior to LOCC metohd for detecting the critical line between two different quantum phases. In order to detect the phase transitions in our model, the ELOCC method is more accurate than LOCC method in the XY model as well as in the one-dimensional transverse field Ising model^25^. Moreover, we should note that if the LOCC convertibility exists, the ELOCC convertibility must exist. That is, in different phases of quantum critical systems, various and complicated local conversion can be expected, will benefit many applications such as to estimate the computational power of different phases.

**Figure 3:**
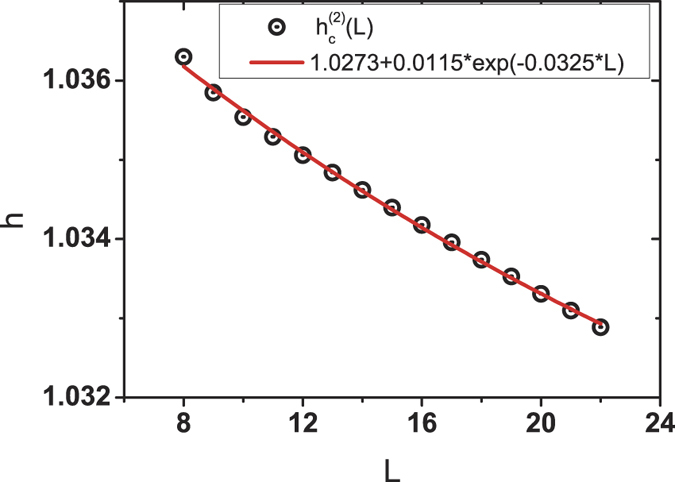
Finite-size scaling analysis. Finite-size scaling analysis of the the critical points 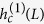 of the second-order phase transition of XY model by the ELOCC convertibility for 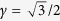.

To get rid of the finite-size effect, we give a scaling analysis of the critical points 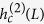 of the second-order phase transition detected by the ELOCC convertibility for 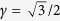 in Fig. 3. With the exponential fitting, it is shown that for infinitely large size *L* → ∞ and 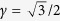, the ELOCC convertibility changes at 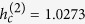, which detects the second-order phase transition of the XY model. The scaling analysis of critical points detected by the LOCC convertibility can be found in ref. 25.

## Discussion

In conclusion, we have investigated the relationship between local convertibility of ground states and quantum phase transitions in XY model. We study the LOCC convertibility by examining the majorization relations and the ELOCC convertibility via Rényi entropy interception. It shows that the boundary of areas which have different LOCC or ELOCC convertibility can be used to detect the critical line for different phases. In Phase 1A, both the LOCC and ELOCC convertibility do not exist. In Phase 1B, the situation is slightly more complicated. There are two areas which has different LOCC convertibility. In most area of Phase 1B, the ELOCC convertibility exists. In Phase 2, there is no LOCC convertibility in the area that nearly the critical point. In the remaining area, both the LOCC and ELOCC convertibility exist. It is obvious that if the LOCC convertibility exists, the ELOCC convertibility must exist. We believe that our method should be applicable in other systems similar as in the XY model. The LOCC proposal in detecting quantum phase transition can play a complementary role to the ELOCC method, which will help to exploit the rich features of local conversion of ground states of quantum critical systems. This study will enlighten extensive studies of quantum phase transitions from the perspective of local convertibility, e.g., finite-temperature phase transitions and other quantum many-body models.

We remark that the majorization relation is necessary and sufficient for the LOCC convertibility^28^ and Renyi entropy interception is a necessary and sufficient condition for the ELOCC convertibility^38^,^39^ between two bipartite pure quantum states. In applying those relations to a ground state of many-body system, it is important to divide the system into bipartitions to obtain the bipartite pure states. In our study of phase diagram of the spin chains, we can show that the local convertibility identified by majorization relation can demonstrate clearly different quantum phases by some bipartitions of the ground state.

## Methods

### Criterions for LOCC and ELOCC convertibility

As showed in ref. 22, we consider a system with an adjustable external parameter *h*, which partitioned into two parties, Alice and Bob, and operated by ELOCC or LOCC. Let |*G*(*g*)〉_*AB*_ be the ground state of the system when the parameter is set as *h*. Given the infinitesimal Δ, the necessary and sufficient condition for ELOCC between |*G*(*h*)〉_*AB*_ and |*G*(*h* + Δ)〉_*AB*_ is given by the inequality *S*_*α*_(*ρ*_*A*_(*h*)) ≥ *S*_*α*_(*ρ*_*A*_(*h* + Δ)) for all values of *α*, where *S*_*α*_(*ρ*_*A*_) is the Rényi entropy of the reduced state of Alice *ρ*_*A*_ = *Tr*_*B*_(|*G*(*h*)〉_*AB*_〈*G*(*h*)|) (called the entanglement Rényi entropy of |*G*(*h*)〉_*AB*_) defined as^23^


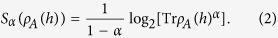


**Figure 4:**
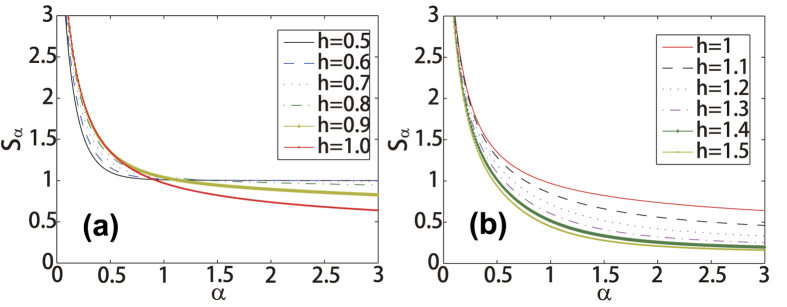
The entanglement Renyi entropy for the ground state. The entanglement Rényi entropy for the ground state of the transverse field Ising model versus *α*. The Rényi entropies are intercepted as (**a**) *h* ≤ 1, while they are non-intercepted as (**b**) *h* ≥ 1, which gives a physical significance to QIP from quantum transitions.

When *α* → 1, the Rényi entropy tends to the von Neumann entropy. In brief, there are two different behaviors for entanglement Rényi entropies of two states neighboring states |*G*(*h*)〉_*AB*_ and |*G*(*h* + Δ)〉_*AB*_. The transverse field Ising model with critical point *h* = 1 is illustrated as an example in Fig. 4: (*a*) If entanglement Rényi entropies are crossing, those two states can not be locally transferred to each other by ELOCC; (*b*) If there is no crossing, a state with higher entanglement can be locally transferred to the lower entanglement one by ELOCC^21^. These results can be applied to study the quantum critical phenomena, i.e. when a quantum phase transition occurs, the local transformation property of the ground state wave function changes as well as the different quantum phases boundaries can be determined by the behavior of entanglement Rényi entropies of ground states^21^,^22^. Referring the scaling analysis in ref. 21 phase transition point obtained by this proposal tends to 0.9940.

On the other hand, the majorization relations provide a necessary and sufficient condition for the LOCC convertibility. Using the Schmidt decomposition theorem^40^, the ground state can be written as 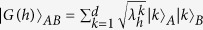. Then, we have that if and only if the majorization relations


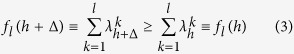


are satisfied for all 1 ≤ *l* ≤ *d* (expressed as 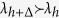, state |*G*(*h* + Δ)〉 can be transformed with 100% probability of success to |*G*(*h*)〉 by LOCC. Otherwise, these two states are incomparable, i.e., |*G*(*h* + Δ)〉 cannot be converted to |*G*(*h*)〉 by LOCC, and vice versa. In ref. 25, it provides a clear description of the local convertibility of the ground states of the transverse field Ising model: in the region 0 < *h* < 0.9940 within the ferromagnetic phase, neither LOCC nor ELOCC convertibility exist; in the region *h* > 1.1318 within the paramagnetic phase, both LOCC and ELOCC convertibility exist; and in the small interval 0.994 < *h* < 1.1318 around the critical point *h* = 1, merely the ELOCC convertibility exists.

### Analytical results for infinite spin chain

For the thermal ground state of XY model written as Equation (1), the reduced density matrix for two nearby spins at *i* and *i* + 1 has been obtained in ref. 41 as


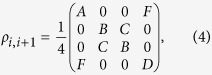


where 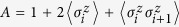, 
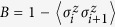
, 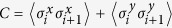, 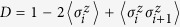 and 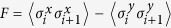. In the thermal limit *T* → 0, we have 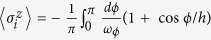, 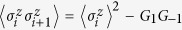, 
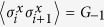
 and 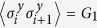 where


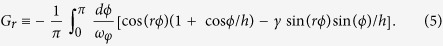


The eigenvalues of the reduced matrix (4) against parameter *h* for *γ* = 0.6 are shown in Fig. 1(b). With the analytical results of the thermal ground state for the infinite long spin chain, the ELOCC convertibility and LOCC convertibility of the ground state of the XY model at zero temperature are presented in Fig. 1(d,f), respectively.

## Additional Information

**How to cite this article**: Liu, S.-Y. *et al*. Phase diagram of quantum critical system via local convertibility of ground state. *Sci. Rep*. **6**, 29175; doi: 10.1038/srep29175 (2016).
